# *Mycobacterium fortuitum* as a cause of peritoneal dialysis-associated peritonitis: case report and review of the literature

**DOI:** 10.1186/1471-2369-13-35

**Published:** 2012-06-08

**Authors:** Simon H Jiang, Darren M Roberts, Andrew H Dawson, Meg Jardine

**Affiliations:** 1 Department of Renal Medicine, Concord Repatriation and General Hospital, Concord, New South Wales, Australia; 2 School of Medicine, University of Queensland, Herston, Queensland, Australia; 3 School of Medicine, University of Western Sydney, Penrith, New South Wales, Australia; 4 Renal & Metabolic Division, The George Institute for Global Health, Camperdown, New South Wales, Australia

**Keywords:** Mycobacterium fortuitum, Peritoneal dialysis, Peritonitis, Treatment

## Abstract

**Background:**

Peritoneal dialysis-associated peritonitis (PD-peritonitis) due to *Mycobacterium spp* is uncommon. Non-tuberculous *Mycobacterium* (NTB) PD-peritonitis can present in a similar fashion to more common causes of bacterial PD-peritonitis. We describe the first reported case of multiresistant *Mycobacterium fortuitum* PD-peritonitis in an Australian patient.

**Case presentation:**

A 38 year-old woman developed mild PD-peritonitis during an overseas holiday. Treatment was complicated by delayed diagnosis, requirement for special investigations, treatment with multiple antibiotics, and conversion to haemodialysis following removal of her Tenckhoff catheter.

**Conclusion:**

This case demonstrates the diagnostic yield of pursuing further investigations in cases of initially culture-negative, problematic PD-peritonitis. A systematic review of the literature identified only 17 reports of *M. fortuitum* PD-peritonitis. Similar to our case, a delay in microbiological diagnosis was frequently noted and the Tenckhoff catheter was commonly removed at the time of diagnosis. The type and duration of antibiotic therapy also varied widely so the optimum treatment appears to be poorly defined.

## Background

Peritoneal dialysis-associated peritonitis (PD-peritonitis) due to *Mycobacterium spp* is uncommon, particularly in patients receiving treatment in developed countries. Non-tuberculous *Mycobacterium* (NTB) PD-peritonitis can present in a similar fashion to more common causes of bacterial PD-peritonitis. However, treatment is often complicated by apparent culture negative dialysate effluent, prolonged treatment courses requiring multiple antibiotics and Tenckhoff catheter removal requiring a change to haemodialysis. We describe the first case of *M. fortuitum* PD-peritonitis in an Australian patient, requiring Tenckhoff catheter removal and permanent conversion to haemodialysis. The literature is reviewed regarding the presentation and treatment of this condition.

## Case presentation

A 38 year-old woman with end-stage renal disease due to Factor-H deficient haemolytic uraemic syndrome had been treated with peritoneal dialysis for 6 months without complication. The patient practiced continuous ambulatory peritoneal dialysis with four exchanges per day of two litres of 2.5% dextrose, each of 4 hour duration. The patient had no prior comorbidities nor history of significant infections. She embarked on a cruise ship holiday in the Pacific. Three days after arriving in North America, she developed nausea which progressed over one week to include mild abdominal pain and diarrhoea. Although her dialysate effluent was cloudy, the mildness of her symptoms prompted the patient to continue the final two weeks of her holiday without any antibacterial treatment. She had no exit site symptoms at any point and reported adherence to her aseptic technique training. Upon return to Australia she promptly presented to hospital.

On presentation to hospital she was febrile (38°C) and tachycardic (108 beats/minute) with a blood pressure of 112/85 mmHg. On examination she had mild abdominal distension and right iliac fossa tenderness. Bowel sounds were present and the Tenckhoff catheter exit site was unremarkable. The peritoneal fluid was turbid with >100 x10^6^/mL leukocytes, predominantly neutrophils.

**Figure 1:**
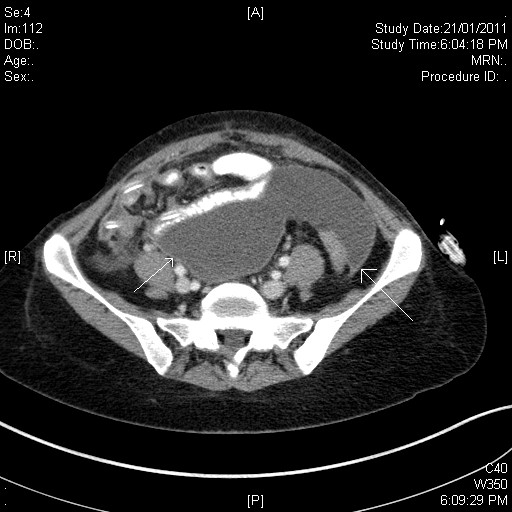
Computerised tomography of the abdomen, demonstrating multiple loculations with thin high attenuation rims.

The peritoneal dialysate cultures were initially reported as culture negative and the patient was treated empirically with intraperitoneal vancomycin and gentamicin. After an early clinical improvement she developed a recurrence of fever and abdominal pain four days after admission. An abdominal computerised tomography scan demonstrated multiple loculations with thin high attenuation rims (Figure 1.). Concurrently, the initial dialysate effluent samples grew gram-positive bacilli after four days consistent with a rapidly-growing non-tuberculous mycobacterium. Gene sequencing by polymerase chain reaction of the isolate identified it to be *M. fortuitum*. Subsequent antibiotic susceptibility testing demonstrated sensitivity to amikacin and ciprofloxacin, intermediate resistance to cefoxitin, and resistance to clarithromycin, doxycycline, imipenem and trimethoprim/sulfamethoxazole.

The patient underwent surgical removal of the Tenckhoff catheter, disruption of loculations and lavage of the peritoneal space. Her subsequent antibiotic regimen of oral moxifloxacin, clarithromycin and doxycycline was complicated by the development of intolerable nausea. She was then established on oral moxifloxacin and linezolid for six months with a sustained clinical response. Treatment duration was empirically determined. After completion of her course of antibiotics re-insertion of the Tenckhoff catheter was attempted but abandoned after multiple intraperitoneal adhesions were found.

## Literature review

We conducted a systematic review of the literature of *M. fortuitum* PD-peritonitis. 17 reports were identified occurring in 12 males and 5 females aged between 15 and 83 years (Table 1.). The geographic spread included reports from North America (8 patients), Asia (4 patients), Europe (3 patients) and the Middle East (2 patients) with none from Australia.

**Table 1:** **Published reports of*M fortuitum*PD-peritonitis**

**Location of the report**	**Patient demographics**	**Time from admission to positive culture**	**Sensitivity**	**Directed Treatment**	**Tenckhoff removed**	**Comment**
Houston, USA [1]	15 y/o male	4 days	S – Amikacin, trimethoprim/sulfamethoxazole, cefoxitin	IV Amikacin and IV cefoxitin. (^c^)	Yes	Abscess drainage required
			R- doxycycline			
Portland, USA [2]	57 y/o male	7 days	Not reported	PO Erythromycin, IV cephapirin, IV tobramycin.(6 days)	Yes	Catheter leak. No abdominal pain. Patient died at 6 days.
Copenhagen, Denmark [3]	35 y/o male	11 days	S – Amikacin, tobramycin, erythromycin, trimethoprim-sulfamethoxazole	IV Amikacin, IV methicillin (^c^)	Yes	In renal transplant perioperative period.
Cleveland, USA [4]	32 y/o male	6 days	S – Amikacin, tetracycline	Amikacin, tetracycline (1 month); ciprofloxacin (^c^, ^d^)	Yes	Reduced aural acuity after amikacin.
			R – gentamicin, tobramycin, erythromycin, trimethoprim/sulfamethoxazole			
Hong Kong, China [5]	65 y/o male	-	Not reported	PO Levofloxacin, PO clarithromycin (12 months)	No	
Seoul, Korea [6]	54 y/o female	7 days	R – isoniazid, rifampicin, ethambutol, streptomycin	IV Ceftizotime, IV amikacin (^c^)	Yes	
Louisiana, USA^b^[7]	71 y/o male	7 days	Not reported	IP Amikacin and cefoxitin (3 weeks); PO doxycycline and rifampicin (3 months)	Yes	
	83 y/o female	4 days	Not reported	Ciprofloxacin and clofazimine (^c^, ^d^)	?	
Madrid, Spain [8]	42 y/o male	11 days	Not reported	IV Amikacin and PO doxycycline (20 days)	Yes	
	40 y/o male	8 days	Not reported	IV Amikacin and PO doxycycline (1 month)	Yes	
Washington, USA [9]	33 y/o male	7 days	S – amikacin, kanamycin, clarithromycin, cefmetazole, imipenem, ciprofloxacin, ofloxacin, azithromycin	PO Clarithromycin and PO trimethoprim/sulfamethoxazole (6 months)	Yes	
	71 y/o female	4 days	R – tobramycin, erythromycin, doxycycline, minocycline, cefoxitin	IV Amikacin, PO clarithromycin, PO trimethoprim/sulfamethoxazole (3 months)	Yes	
			S – clarithromycin, cefmetazole, imipenem, sulfisoxazole, ciprofloxacin, ofloxacin			
			I – amikacin, cefoxitin			
Saudi Arabia [10]	45 y/o female	7 days		PO Isoniazid, rifampicin, ethambutol. (6 months)	Yes	
Nashville, USA [11]	16 y/o male	12 days		PO Ciprofloxacin, PO trimethoprim/sulfamethoxazole (^c^)	Yes	Perihepatic collection
Kfar-Saba, Israel [12]	65 y/o male	4 days		Minocycline (1 month^d^)	Yes	Also bacteraemia
Tokyo, Japan [13]	50 y/o male	3 weeks	Not reported	Amikacin, sulbactam (^c^,^d^)	Yes	
Singapore, Singapore ^b^[14]	78 y/o female	8 days	S – tobramycin, linezolid, clarithromycin, amikacin	IP amikacin, PO ciprofloxacin (3 months)	No	Patient died at 2 months from malignancy

^a^This publication also reported on a suspected case of PD-peritonitis and two confirmed cases of Tenckhoff catheter exit site infections due to *M. fortuitum*.

^b^This publication also listed a second (male) patient with *M. fortuitum* PD-peritonitis in a table, but described the patient to be a female with *M. abscessus* PD-peritonitis in the text. Due to inconsistency in the report, this case was excluded.

^c^Duration of therapy not known.

^d^Route of administration not known.

*IP* intraperitoneal, *PO* per oral, *IV* intravenous.

The duration of symptoms prior to hospital presentation was incompletely reported and a delay in microbiological diagnosis was commonplace (median 7 days, interquartile range 4-8 days). Intra-abdominal loculations and collections occurred in 2 previous reports*.* The Tenckhoff catheter was removed in all but two reported cases and mostly at the time of diagnosis. The duration of antibiotic therapy varied widely (between 1 and 12 months), so the optimum treatment appears to be poorly defined. In our patient the 6 month duration was chosen based on typically reported dosing periods. Antibiotic sensitivity by disc diffusion was reported in 10 of the 17 reports. *M. fortuitum* was almost universally sensitive to amikacin, ciprofloxacin, imipenem and clarithromycin. Resistance to cephalosporins, tetracyclines and other macrolides was variable. Similarly, there was marked variability in the antibiotic regimens utilised and the rationale for these choices were incompletely described. The more common antibiotics utilised included amikacin in 7 reports, ciprofloxacin in 4 reports and doxycycline in 3 reports with combination therapy common. Only one death was reported in a patient with *M. fortuitum* PD-peritonitis (Table 1).

## Conclusion

We describe the first case of *M. fortuitum* PD-peritonitis and the third case of any NTM PD-peritonitis in an Australian patient [15,16]. The place and mode of infection remain unclear. Our patient had recently returned from a one month overseas holiday traversing the Pacific, the Caribbean and North America. Given the slow growth rate of *M. fortuitum* her peritoneum may have been inoculated either before leaving Australia, whilst travelling through the Caribbean or North America. The slow growth rate of *M. fortuitum* presumably accounts for the mild and indolent development of symptoms and may be a clinical characteristic of NTM PD-peritonitis. The subacute course may predispose to the chronic sequelae of infection. Our case brings the number of cases with reported intra-abdominal loculations to three in eighteen. This proportion could well be an underestimate with only a minority of reported patients having imaging or persisting with peritoneal dialysis.

The incidence of PD-peritonitis due to *Mycobacterium spp.* in Australia has not been described. On the basis of registry data, it is anticipated to be low given the relatively high cure rates of all cases of PD-peritonitis in Australia after a median duration of treatment of 13 days with primarily vancomycin or cephalosporins with/without gentamicin [17]. There is considerable variability in the proportion of cases of PD-peritonitis that are culture-negative in Australian and New Zealand renal units which may reflect differences in disease prevalence and/or diagnostic and management practices [17].

Guidelines of the International Society of Peritoneal Dialysis (ISPD) recommend that a negative dialysate culture at 3 days in the presence of ongoing clinical evidence of peritonitis calls for specialized cultures for atypical causes of peritonitis [18]. If there is a clinical suspicion of infection with *Mycobacterium spp.*, repeated smears and centrifuge of effluent sediment with a combination of solid- and fluid-medium culture are suggested.

The safety and timing for attempted re-insertion of Tenckhoff catheters after treatment, and technique survival, is incompletely reported. The ISPD guidelines recommend consideration of catheter removal following a diagnosis of Mycobacterial peritonitis although data supporting this recommendation are limited.

Non-tuberculous Mycobacteria are a rare but serious cause of PD-peritonitis with high rates of Tenckhoff removal and conversion to haemodialysis. This is the first report of *M. fortuitum* PD-peritonitis reported in Australia and the diagnosis was prompted by inadequate response to empiric antibiotics and confirmed by special investigations that are uncommonly utilised in most cases of PD-peritonitis.

## Consent

Written informed consent was obtained from the patient for publication of this case report and any accompanying images. A copy of the written consent is available for review by the Editor-in-Chief of this journal.

## Abbreviations

ISPD, International Society of Peritoneal Dialysis; IP, Intraperitoneal; IV, Intravenous; NTB, Non-tuberculous Mycobacterium; PD-peritonitis, Peritoneal dialysis-associated peritonitis; PO, Per oral.

## Competing interests

The authors declare that they have no competing interests.

## Authors’ contributions

SJ, DR and MJ were involved with the clinical management of the patient. SJ and DR drafted the manuscript. All authors revised and approved the final manuscript.

## Pre-publication history

The pre-publication history for this paper can be accessed here:

http://www.biomedcentral.com/1471-2369/13/35/prepub

## References

[B1] LaRocco MT Mortensen JE Robinson A Mycobacterium fortuitum peritonitis in a patient undergoing chronic peritoneal dialysis Diagn Microbiol Infect Dis 1986 4 2 161 164 10.1016/0732-8893(86)90151-3 3956137

[B2] Pulliam JP Vernon DD Alexander SR Nontuberculous mycobacterial peritonitis associated with continuous ambulatory peritoneal dialysis Am J Kidney Dis 1983 2 6 610 614 6846333 10.1016/s0272-6386(83)80040-7

[B3] Kolmos HJ Brahm M Bruun B Peritonitis with Mycobacterium fortuitum in a patient on continuous ambulatory peritoneal dialysis Scand J Infect Dis 1992 24 6 801 803 10.3109/00365549209062468 1287816

[B4] Woods GL Hall GS Schreiber MJ Mycobacterium fortuitum peritonitis associated with continuous ambulatory peritoneal dialysis J Clin Microbiol 1986 23 4 786 788 3700629 10.1128/jcm.23.4.786-788.1986 PMC362838

[B5] Tang S Tang AW Lam WO Successful treatment of Mycobacterium fortuitum peritonitis without Tenckhoff catheter removal in CAPD Perit Dial Int 2003 23 3 304 305 12938837

[B6] Choi CW Cha DR Kwon YJ Mycobacterium fortuitum peritonitis associated with continuous ambulatory peritoneal dialysis Korean J Intern Med 1993 8 1 25 27 8268143 10.3904/kjim.1993.8.1.25 PMC4532079

[B7] White R Abreo K Flanagan R Nontuberculous mycobacterial infections in continuous ambulatory peritoneal dialysis patients Am J Kidney Dis 1993 22 4 581 587 8213799 10.1016/s0272-6386(12)80932-2

[B8] Soriano F Rodriguez-Tudela JL Gomez-Garces JL Two possibly related cases of Mycobacterium fortuitum peritonitis associated with continuous ambulatory peritoneal dialysis Eur J Clin Microbiol Infect Dis 1989 8 10 895 897 10.1007/BF01963778 2512136

[B9] Vera G Lew SQ Mycobacterium fortuitum peritonitis in two patients receiving continuous ambulatory peritoneal dialysis Am J Nephrol 1999 19 5 586 589 10.1159/000013524 10575188

[B10] Youmbissi JT Malik QT Ajit SK Non tuberculous mycobacterium peritonitis in continuous ambulatory peritoneal dialysis J Nephrol 2001 14 2 132 135 11411016

[B11] Dunmire RB Breyer JA Nontuberculous mycobacterial peritonitis during continuous ambulatory peritoneal dialysis: case report and review of diagnostic and therapeutic strategies Am J Kidney Dis 1991 18 1 126 130 2063847 10.1016/s0272-6386(12)80303-9

[B12] Hod T Kushnir R Paitan Y Mycobacterium fortuitum infection in continuous ambulatory peritoneal dialysis Clin Nephrol 2008 70 6 546 553 19049716 10.5414/cnp70546

[B13] Kawamoto S Otani K Kawaguchi Y Mycobacterium fortuitum peritonitis associated with CAPD: diagnosis by a molecular biology technique Perit Dial Int 1999 19 6 592 593 10641784

[B14] Renaud CJ Subramanian S Tambyah PA The clinical course of rapidly growing nontuberculous mycobacterial peritoneal dialysis infections in Asians: a case series and literature review Nephrology 2011 16 2 174 179 10.1111/j.1440-1797.2010.01370.x 21272129

[B15] Linton IM Leahy SI Thomas GW Mycobacterium gastri peritonitis in a patient undergoing continuous ambulatory peritoneal dialysis Aust N Z J Med 1986 16 2 224 225 10.1111/j.1445-5994.1986.tb01155.x 3463276

[B16] Jiang SH Senanayake S Talaulikar GS Peritoneal dialysis-related peritonitis due to Mycobacterium smegmatis Perit Dial Int 2011 31 2 215 216 21427257 10.3747/pdi.2010.00074

[B17] Fahim M Hawley CM McDonald SP Culture-negative peritonitis in peritoneal dialysis patients in Australia: predictors, treatment, and outcomes in 435 cases Am J Kidney Dis 2010 55 4 690 697 10.1053/j.ajkd.2009.11.015 20110144

[B18] Li PK Szeto CC Piraino B Peritoneal dialysis-related infections recommendations: 2010 update Perit Dial Int 2010 30 4 393 423 10.3747/pdi.2010.00049 20628102

